# Grapevine rootstock effects on scion sap phenolic levels, resistance to *Xylella fastidiosa* infection, and progression of Pierce's disease

**DOI:** 10.3389/fpls.2013.00502

**Published:** 2013-12-12

**Authors:** Christopher M. Wallis, Anna K. Wallingford, Jianchi Chen

**Affiliations:** USDA-Agricultural Research Service, San Joaquin Valley Agricultural Sciences CenterParlier, CA, USA

**Keywords:** Pierce's disease, *Xylella fastidiosa*, *Vitis vinifera*, rootstock, phenolics

## Abstract

The xylem-limited bacterium *Xylella fastidiosa* (Xf) causes Pierce's disease (PD), an important disease of grapevine, *Vitis vinifera* L. Grapevine rootstocks were developed to provide increased resistance to root disease, but rootstock effects on cane and vine diseases remain unclear. Grapevines that consisted of Cabernet Sauvignon or Chardonnay grafted to 13 different rootstocks were inoculated with Xf and evaluated for PD severity and Xf titer after 6 months. A subset of six rootstock/scion combinations had xylem sap phenolic levels assessed in non-infected and Xf-infected grapevines. Vigor also was analyzed by measuring root lengths and masses. Cabernet Sauvignon grafted to 101-14MG, 1103P, 420A, or Schwarzmann had reduced PD severity compared to Cabernet Sauvignon grafted to 110R, 5BB, or SO4. Chardonnay grafted to Salt Creek or Freedom had reduced PD severity compared to Chardonnay grafted to RS3 or Schwarzmann. Chardonnay grafted to RS3 had greater Xf titer than Chardonnay grafted to 101-14MG, Freedom, or Salt Creek. No other differences in Xf titer among rootstocks were observed. Of the six scion/rootstock combinations which had xylem sap phenolics analyzed, Chardonnay/RS3 had the highest levels of most phenolics whereas Cabernet Sauvignon/101-14MG had the lowest phenolic levels. However, Chardonnay/101-14MG, which had mild PD symptoms, had greater sap levels of caftaric acid than other scion/rootstock combinations. Sap levels of caftaric acid, methyl salicylate, a procyanidin trimer, and quinic acid were greater in Xf-infected vs. non-infected grapevines. Chardonnay on 101-14MG or Salt Creek had greater root mass than Chardonnay on RS3. Cabernet Sauvignon on 101-14MG had greater root mass than Cabernet Sauvignon on 110R. These results identified rootstocks with the capacity for reducing PD symptom progression. Rootstocks also were shown to affect Xf titer, xylem sap phenolic levels, and plant vigor.

## Introduction

Grapevine cultivars are generally grown for specific fruit qualities and are predominately selections of *Vitis vinifera* L., which is propagated throughout the world. Unfortunately, a variety of diseases, such as Pierce's disease (PD), impact *Vitis vinifera* in many warmer regions. PD is caused by a strain of the xylem-limited bacterium *Xylella fastidiosa* Hopkins (Xf), which is thought to have originated in Northern Mexico and the Southeastern United States (Wells et al., 1987). In these places, wild grape species are tolerant of the disease and do not exhibit symptoms when infected with Xf (Keller, 2010).

However, the fruit of North American wild grape species often have undesirable characteristics such a poor berry taste, berry size, and vine growth habit. One solution to impart increased disease tolerance to commercial grape plantings and to keep desired fruit characteristics is to graft desirable grape cultivars to rootstocks improved for disease/pest resistance (Christensen, 2003; Keller, 2010). The use of North American rootstocks to graft to scion of European cultivars to has been credited with saving the European vineyard industry from the root-feeding insect phlloxera (Galet, 1996; Granett et al., 2001). Grafting also can protect from nematodes and soil-dwelling pathogens such as crown gall-causing *Agrobacterium vitis* (Anwar et al., 2002; Keller, 2010). Rootstocks that are considered “resistant” generally have an increased tolerance to pathogen or pest attack through a variety of physical and chemical mechanisms that limit feeding and pathogen progression (Granett et al., 2001; Keller, 2010).

However, the ability of rootstocks to impart increased tolerance against scion diseases is less understood. For some scion diseases affecting grapevines or other grafted plants, different rootstocks have resulted in reduced symptom progression or pathogen titers (Gould et al., 1991; He et al., 2000; Cousins and Goolsby, 2011). Cousins and Goolsby (2011) found the scion/rootstock combination with the greatest pruning weights also had fewer PD symptoms, which could imply that vine vigor improves tolerance to Xf infection. Rootstocks might impact tolerance to pathogen infection by the ability to influence scion vigor and nutrient uptake (Ruhl et al., 1988; Keller et al., 2001). Improved vigor and nutrient uptake could in turn provide greater host resources needed for production of secondary metabolites (including compounds called phenolics) and other components of host defense against pathogens. Presumably, compounds produced by the rootstock also could translocate throughout the scion via xylem sap. This also could impact the growth of Xf and other xylem-limited pathogens.

Previously, xylem sap components were shown to affect bacterial pathogen growth, proliferation, aggregations, and biofilm formation (Cheng et al., 2009; Cruz et al., 2012; Shi et al., 2013). In particular, phenolic compounds have been shown to inhibit growth of Xf *in vitro* (Maddox et al., 2010). Phenolic levels may increase to some degree in response to infection by Xf (Wallis and Chen, 2012; Wallis et al., 2013). This is important because phenolics also have been repeatedly associated with host resistance to bacterial and other diseases (Derckel et al., 1999; Goetz et al., 1999; Hammerschmidt, 2004; Pezet et al., 2004; Gutha et al., 2010; Rusjan et al., 2012).

The objective of this work was to observe whether different rootstocks could affect PD symptom progression, Xf growth, or levels of defense-associated phenolic compounds. Greater phenolic levels could be due to either differences in genotype or vigor, and therefore rootstock vigor was assessed as well. Two different scion cultivars were examined to observe if rootstock effects on PD and Xf were consistent when scion varied. This work elucidated the ability of rootstocks to impart increased tolerance to XF infection and reduce PD symptom progression. Furthermore, the ability of rootstocks to affect xylem sap phenolic levels was observed.

## Materials and methods

### Plant material

Sixteen vines each were obtained from a local nursery for Chardonnay grafted to six commonly utilized rootstocks (Table 1). Likewise, an addition 16 vines each were obtained from a local nursery for Cabernet Sauvignon grafted to 10 different rootstocks (Table 1). All of these grapevines were planted in potting media and arranged in a randomized, complete block design (with two spatial blocks) in a climate-controlled greenhouse that had a 14 h light cycle.

**Table 1:** **Scion and rootstock combinations used in this study, with information about the wild *Vitis* spp. in the background of the rootstocks provided**.

**Scion**	**Rootstock**	**Background**
Cabernet Sauvignon	1103 Paulsen (1103P)	*V. berlandieri* × *V. rupestris*
	110 Richter (110R)^*^	*V. berlandieri* × *V. rupestris*
	101-14 Millardet et de Grasset (101-14)^*^	*Vitis riparia* × *V. rupestris*
	1616 Couderc	*V. solonis* × *V. riparia*
	3309 Couderc	*V. riparia* × *V. rupestris*
	O39-16 (3916)	*V. vinifera* × *V. rotundifolia*
	420A Millardet et de Grasset (420A)	*V. berlandieri* × *V. riparia*
	5BB [Kober]	*V. berlandieri* × *V. riparia*
	Schwarzmann	*V. riparia*
	SO4	*V. berlandieri* × *V. riparia*
Chardonnay	110 Richter (110R)^*^	*V. berlandieri* × *V. rupestris*
	101-14 Millardet et de Grasset (101-14)^*^	*Vitis riparia* × *V. rupestris*
	Freedom	*V. champinii* × *V. solonis* × *V. riparia*
	RS-3 (RS3)^*^	SC × Schwarzmann
	Salt Creek [Ramsey] (SC)^*^	*V. champinii*
	Schwarzmann	*V. riparia*

Alternative rootstock names are given in brackets, and abbreviations used are given by parenthesis. Scion/rootstock combinations with an

* were selected for xylem sap phenolic and vigor analyses.

### Evaluation of PD and Xf

In April 2012, eight plants of each scion/rootstock combination were thrice inoculated ~5 cm above the rootstock with the Stag's Leap strain of Xf using the pin-prick method described by Hopkins (2001). The remaining vines were mock-inoculated with water. In brief, Xf grown on periwinkle wilt agar media was suspended in 2 mL of water (for 1.0 × 10^8^–1.0 × 10^9^ CFUs/mL), and a 23-gauge needle was used to prick holes on the stem (Hopkins, 2001). These holes then had droplets of bacterial suspension placed on them, which was absorbed to inoculate the plant. Two weeks after initial inoculation, a second inoculation was conducted to improve infection success (Wallis and Chen, 2012).

Inoculated grapevines were assessed for PD symptom severity in October, 6 months after initial inoculation treatment, using a 0–5 scale, with “0” representing no disease symptoms, “1” less than 25% diseased leaf area, “2” between 26 and 50% diseased leaf area and minor dwarfing (reduced growth and internodes), “3” between 51 and 75% diseased leaf area and moderate dwarfing, “4” over 76% diseased leaf area and severe dwarfing, and “5” representing dead plants (Wallis and Chen, 2012).

Bacterial titers were assessed according to the methods described by Chen et al. (2005). Xf DNA was extracted from 100 mg of pulverized petiole tissue collected at the end of the experiment using a DNeasy Plant Mini Kit (Qiagen, Valencia, CA). Titers were then determined by qPCR using primers from Chen et al. (2005), SYBR Green Mastermix (BioRad, Hercules, CA), and performing the qPCR reaction on an Option 2 Real Time PCR System (BioRad). Standard curves of known amounts of Xf DNA were used to convert calculated titers to ng Xf DNA/g fresh weight.

### Phenolic analyses

Although ~10 g of shoot samples from all plants were collected and kept frozen, a subset of scion/rootstock combinations was selected for phenolic analyses due to the difficulty of obtaining adequate xylem sap and performing chemical analyses. The six chosen grapevine scion rootstock combinations were selected based on the clearest differences in PD severity and/or the having the same rootstock in common for each scion cultivar. The following were thus used for phenolic analyses as well as to determine differences in root vigor: Chardonnay grafted to 101-14, 110R, RS3, and SC; and Cabernet Sauvignon grafted to 101-14 and 110R.

Sap was collected from the selected grapevines and analyzed for phenolic content using methods described in Wallis et al. (2008) and Wallis and Chen (2012). In brief, at the end of the experiment ~10 g of green shoot segment were collected roughly 20 cm from the initial inoculation site, placed into 15 mL centrifuge tubes, flash frozen in liquid nitrogen, and stored at −20°C until processed. After a brief time to thaw, shoot segments were prepared for xylem sap extraction by peeling away the outer bark and phloem layer. Shoot segments were then cut to fit into two 1.5 mL microcentrifuge tubes that had ~10 glass beads on the bottom. These tubes were centrifuged at 15,000 X g for 10 min held at 4°C. Average sap yield was 50–100 μL per tube (total yield of ~100–200 μL), although not every plant had sufficient xylem sap recovered.

High-performance liquid chromatography (HPLC) was performed on xylem sap according to the methods of Wallis and Chen (2012). In brief, a Shimadzu (Columbia, MD, USA) LC-20AD system equipped with a Shimadzu XR-ODS C18 column and a PDA-20A photodiode array detector ran a binary gradient using 0.2% acetic acid (Sigma-Aldrich, St. Louis, MO, USA) in water for Solvent A and 0.2% acetic acid in methanol (Thermo-Fisher Scientific, Pittsburgh, PA, USA) for Solvent B. Quantification of compound peaks was made at 280 nm, with no peaks observed that did not absorb at least partially at 280 nm. Sap samples were diluted 1:1 (v/v) in water and a volume of 50 μL was injected for HPLC analyses. Compound peaks were previously identified by matching UV/Vis spectra and masses obtained with a Shimadzu LCMS-2020 as performed by Wallis and Chen (2012). Commercial standards were used for definitive identifications by retention time matching. Standard curves were derived using catechin, quercetin, caftaric acid, or ferulic acid (all from Sigma) to convert identical and similar compounds into μg/mL amounts (Wallis and Chen, 2012).

### Root mass and length measurements

At the end of the experiment, the grapevines selected for phenolic analyses (Chardonnay on 101-14, 110R, ES3, and SC; and Cabernet Sauvignon on 101-14 and 110R) also had roots isolated by cutting and removal all aboveground portions of the plant at the soil surface. Potting media was removed from around the roots, the roots were washed, and then had root length measured. Roots were then placed into a dryer for 7 days at 38°C, after which dry weights were recorded.

### Statistical analyses

SPSS ver. 19.0 (IBM, Armonk, NY, USA) was used for all data analyses with α set to 0.05. The SPSS EXPLORE feature was used to check for normality via residual plots for Xf titer and phenolic data. Due to disease severities being on a 0–5 scale, the non-parametric Kruskal–Wallis test was used to determine overall differences in disease severity due to rootstock for each scion cultivar, with Mann–Whitney *U*-tests were used to identify differences pairwise. Mann–Whitney *U*-tests also were used to observed if differences due to scion existed in the three rootstocks common to both (101-14, 110R, and Schwarzmann).

Multivariate analyses of variance (MANOVA), with Pillai's trace as the test statistic, were performed to examine the effects of infection status and/or scion/rootstock combination on overall phenolic content of sap (block effects were not significant and removed from analyses). Following MANOVA, follow-up ANOVA on individual compounds and subsequent *post-hoc* LSD tests were performed to separate scion/rootstock combinations. In total, three different MANOVA analyses were performed. The first analyzed non-infected grapevines only to observe effects of scion/rootstock on constitutive levels of sap phenolics. The second analyzed infected grapevines only to observe effects of scion/rootstock on induced levels of sap phenolics. Finally, all grapevines were included in MANOVA to observe the effects of infection status, scion/rootstock combination, and the interaction of both on sap phenolic levels.

Analyses of variance (ANOVA), with rootstock as the independent variable, were used to determine differences in bacterial titer, length to root mass ratios and total root mass, followed by *post-hoc* Least Significant Difference (LSD) mean separation tests when required. Pairwise *t*-tests were used to observe differences due to scion in rootstocks common to each (110R, 101-14, and “Schwarzmann”).

Spearman ρ correlations were performed to find correlations between symptom severity scores with Xf titer, root mass, or levels of individual phenolic compounds found in xylem sap. Pearson correlations were used to find associations between Xf titer, levels of sap compounds, and root mass.

## Results

### Rootstock effects on PD symptoms

**Figure 1:**
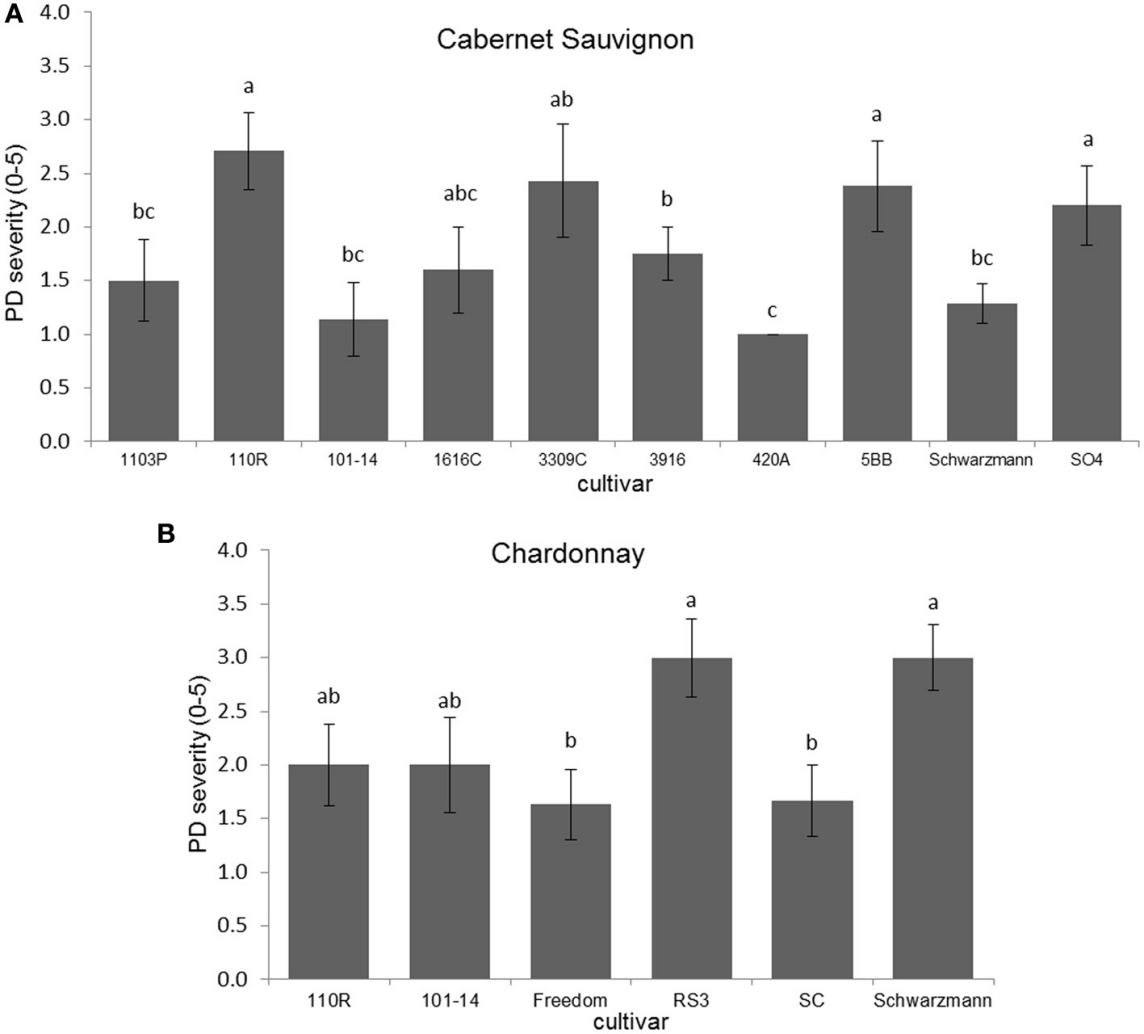
**Disease severity of (A) Cabernet Sauvignon or (B) Chardonnay scion on various rootstock cultivars**. Letters represent differences pairwise by Mann–Whitney *U*-tests. Bars indicate SE. See text for full descriptions of rootstocks.

Disease severity was significantly different due to rootstock for Cabernet Sauvignon (*H* = 18.423; *P* = 0.031; *N* = 67), with plants on 110R rootstock having greater PD severity than those on Schwarzmann, 101-14, 420A, 1103P, and O39-16 (Figure 1A). Disease severity also was significantly different due to rootstock for Chardonnay (Kruskal–Wallis *H* = 11.878, *P* = 0.036; *N* = 39), with plants having RS3 and Schwarzmann rootstocks having more severe PD symptoms than plants having SC or Freedom rootstocks (Figure 1B). Comparing scion effects on PD severity when on the same rootstock (101-14, 110R, or Schwarzmann), only when Schwarzmann was used as a rootstock did PD severity differ due to cultivar (Mann–Whitney *U* = 2.000; *P* = 0.003; *N* = 14), with greater severity in Cabernet Sauvignon than Chardonnay.

### Rootstock effects on Xf titer

**Figure 2:**
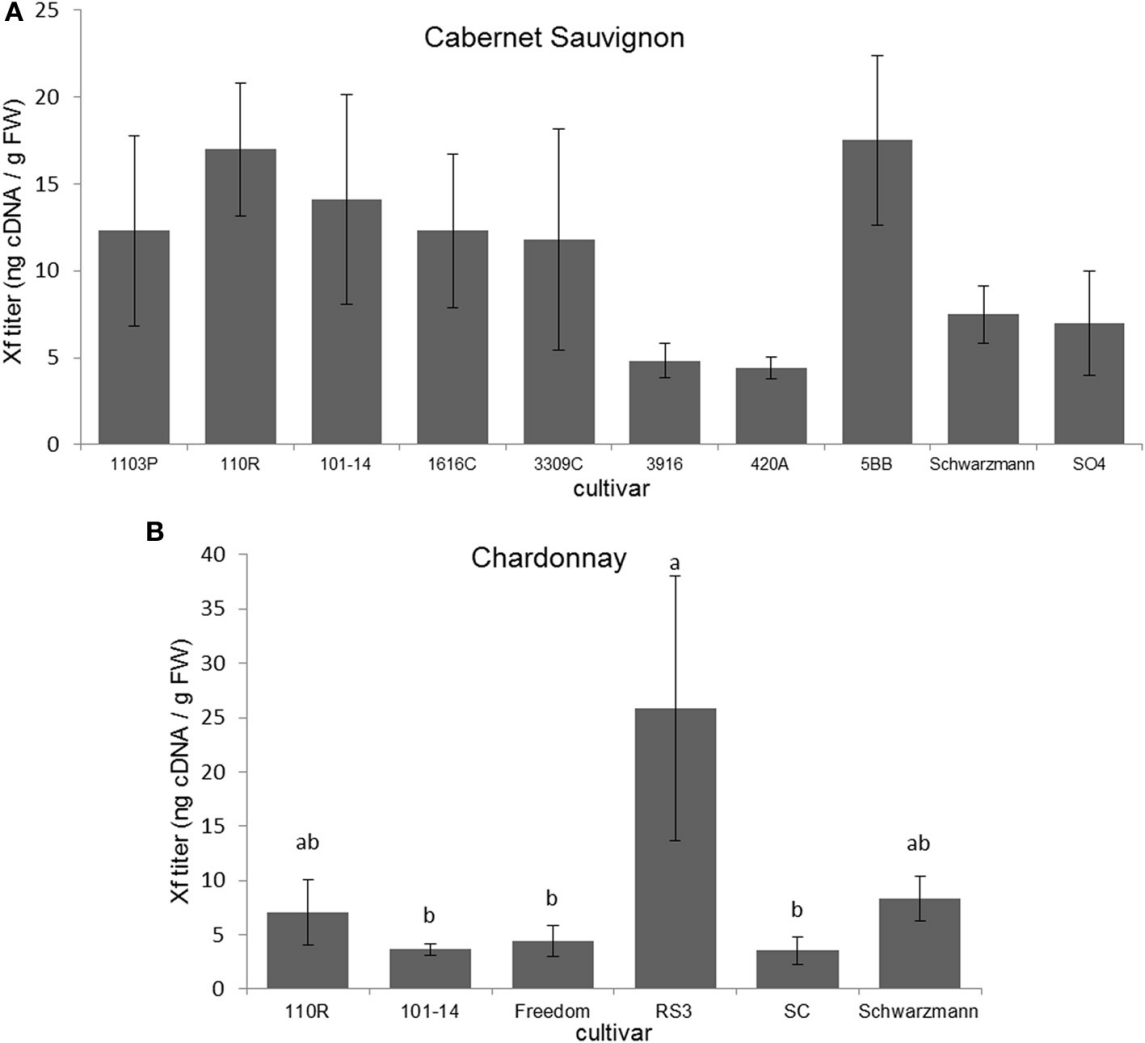
**Xf titer found present in (A) Cabernet Sauvignon or (B) Chardonnay scion on various rootstock cultivars**. Letters represent differences pairwise by LSD tests. Bars indicate SE. See text for full descriptions of rootstocks.

Xf titers did not significantly differ among Cabernet Sauvignon grapevines grafted to different rootstocks (*F* = 1.152; *P* = 0.348; *N* = 55) (Figure 2A). Xf titers were significantly greater (*F* = 3.013; *P* = 0.030; *N* = 30) in Chardonnay grapevines grafted to RS3 than those grafted to 101-14, Freedom, or SC (Figure 2B). There were no significant differences (*P* > 0.05) in Xf titer due to scion when comparing Cabernet Sauvignon and Chardonnay grafted to the same rootstock (101-14, 110R, or Schwarzmann).

For Cabernet Sauvignon grapevines there was no significant relationship between disease severity and Xf titer (ρ = −0.118; *P* = 0.389; *N* = 55). However, for Chardonnay grapevines, disease severity ratings were positively associated with Xf titer (Spearman ρ = 0.435; *P* = 0.016; *N* = 30).

### Selected rootstock effects on xylem sap biochemistry

Twenty phenolic compounds were identified and quantified in xylem sap (Table 2).

**Table 2:** **Compounds quantified from xylem sap for this study, with criteria for putative identifications provided**.

**Compound**	**Retention time**	**Absorbance maxima**	**Mass**
Caftaric acid^*^	5.858	275, 308	312
Catechin^*^	8.23	277	290
Epicatechin gallate^*^	10.093	277	442
Epicatechin^*^	11.094	277	578
Flavonoid glycoside 1	12.758	277	468
Flavonoid glycoside 2	17.175	277	778
Methyl salicylate dimer	8.828	278	600
Procyanidin B isomer 1	4.423	276	594
Procyanidin B isomer 2	5.143	275	594
Procyanidin B isomer 3	10.38	276	730
Procyanidin B isomer 4	14.422	277	578
Procyanidin B1^*^	7.281	277	578
Procyanidin B2^*^	9.32	277	578
Procyanidin C isomer 1	3.679	271	866
Procyanidin C isomer 2	7.65	277	866
Procyanidin C isomer 3	13.889	279	906
Procyanidin C isomer 4	15.704	278	906
Procyanidin C1^*^	10.72	277	866
Quinic acid dimer	1.658	265	380
Quinic acid^*^	2.366	265	192

Compounds with an

* were confirmed by matching retention times with those of obtained commercial standards.

Constitutive levels of sap phenolics, i.e., levels from non-inoculated grapevines, were affected by scion/rootstock combination according to MANOVA (Pillai's Trace Δ = 3.534; *F* = 1.687; *P* = 0.011; *N* = 35). Subsequent ANOVA analyses revealed that levels of 15 of 20 phenolic compounds were different due to scion/rootstock combination, with only the two procyanidin B isomers, procyanidin C isomer 2, flavonoid glycoside 2, and quinic acid dimer not significantly (*P* > 0.05) affected by rootstock combination (Table 3). LSD tests revealed that most phenolic levels were greater in Chardonnay/RS3 than all other combinations, whereas phenolic levels were generally lower in Cabernet Sauvignon/101-14 than other combinations (Table 3).

**Table 3:** **Mean (±SE) sap levels (μg/mL) of phenolic compounds from non-infected grapevines with different scion/rootstock combinations**.

**Compound**	**Cabernet Sauvignon**		**Chardonnay**				***F***
	**101-14**	**110R**	**101-14**	**110R**	**RS3**	**SC**	
Caftaric acid	35.3±8.3 b	32.9±4.5 b	77.6±16.4 a	38.7±8.0 b	49.6±7.9 b	36.7±7.3 b	3.031^*^
Catechin	292±42 b	423±76 b	408±70 b	489±30 ab	658±66 a	413±97 b	3.279^*^
Epicatechin	241±86 b	524±132 ab	549±166 ab	646±40 a	790±84 a	568±91 a	3.538^*^
Epicatechin gallate	155±24 c	246±48 bc	284±36 ab	313±46 ab	357±37 a	305±40 ab	3.661^*^
Flavonoid glycoside 1	16.8±2.4 c	42.5±11.0 bc	72.0±32.8 abc	72.4±10.9 abc	126±33 a	79.1±16.9 ab	3.592^**^
Flavonoid glycoside 2	24.4±11.8	91.8±31.0	48.0±13.4	45.6±3.2	57.1±13.9	30.3±5.1	2.285
Methyl salicylate	18.5±3.3 c	26.5±7.0 bc	40.4±1.9 ab	38.6±5.4 ab	42.7±5.3 a	26.7±4.0 bc	4.010^**^
Procyanidin B isomer 1	64.0±9.9	77.4±8.1	64.5±11.6	76.4±6.5	87.3±9.5	78.4±12.4	0.772
Procyanidin B isomer 2	65.8±16.5	79.5±17.0	89.6±17.1	197±80	116±20	79.1±14.9	2.459
Procyanidin B isomer 3	142±24 b	255±54 a	281±37 a	295±11 a	343±26 a	296±41 a	4.088^**^
Procyanidin B isomer 4	34.0±6.5 d	70.0±13.1 cd	169±45 ab	119±10 bc	196±30 a	126±24 bc	7.196^***^
Procyanidin B1	177±29 c	319±67 bc	274±33 bc	327±17 bc	543±52 a	418±82 ab	5.287^***^
Procyanidin B2	113±43 c	230±55 bc	319±59 abc	294±65 abc	439±59 a	350±93 ab	3.212^*^
Procyanidin C isomer 1	53.1±6.8 c	70.3±11.8 bc	68.1±9.5 bc	86.4±7.1 ab	109±9 a	96.4±14.7 ab	3.980^**^
Procyanidin C isomer 2	122±16	194±50	150±23	214±41	301±43	226±60	2.186
Procyanidin C isomer 3	56.2±12.9 c	180±43 ab	142±45 abc	185±42 ab	223±40 a	107±18 bc	3.732^**^
Procyanidin C isomer 4	25.5±7.1 b	69.1±16.6 b	85.9±15.4 ab	88.8±9.0 ab	132±38 a	66.6±8.1 b	3.099^*^
Procyanidin C1	112±27 b	225±57 a	250±46 a	257±13 a	291±25 a	275±43 a	3.198^*^
Quinic acid	25.0±3.3 b	26.4±3.1 b	23.0±5.8 b	43.2±10.7 a	22.5±3.7 b	15.1±2.8 b	3.494^*^
Quinic acid dimer	25.0±3.2	31.4±2.4	28.0±4.9	36.5±8.6	37.4±4.2	24.2±3.2	1.974

The F-test statistic is provided. Different letters next to the means indicate significant differences as determined by LSD tests.

* P < 0.05;

** P < 0.01;

*** P < 0.001.

Induced levels of sap phenolics, i.e., levels from Xf-infected grapevines, also were affected by scion/rootstock combination according to MANOVA (Δ = 3.636; *F* = 1.599; *P* = 0.025; *N* = 33). However, only procyanidin B isomer 2 (*F* = 3.292; *P* = 0.019) and caftaric acid (*F* = 3.388; *P* = 0.017) significantly differed due to scion/rootstock combination according to follow-up ANOVAs. Procyanidin B isomer 4 occurred at lower levels in both Cabernet Sauvignon combinations than Chardonnay grafted to 101-14, RS3, or SC. Caftaric acid occurred at lower levels in both Cabernet Sauvignon combinations and Chardonnay grafted to 110R than Chardonnay grafted to 101-14 or RS3.

MANOVA analyses on both non-infected and infected plants revealed significant effects on sap phenolic levels due to scion/rootstock combination (Δ = 2.502; *F* = 2.053; *P* < 0.001; *N* = 68) and infection status (Δ = 0.795; *F* = 7.188; *P* < 0.001; *N* = 68). The interaction between scion/rootstock combination and infection status was non-significant (Δ = 1.849; *F* = 1.203; *P* = 0.136; *N* = 68). Subsequent ANOVA analyses revealed that only six compound were not significantly affected by scion/rootstock combination: two procyanidin B isomers, two procyanidin C isomers, flavonoid glycoside 2, and quinic acid dimer (Table 4). Cabernet Sauvignon grapevines generally had lower levels of many of the phenolics than Chardonnay grafted on RS3 or SC (Table 4). Infection status significantly (*P* < 0.05) affected levels of procyanidin C isomer 1 (with greater levels in Xf-infected plants), procyanidin C isomer 3 (with greater levels in non-infected plants), procyanidin B isomer 3 (with greater levels in non-infected plants), caftaric acid (with greater levels in Xf-infected plants), methyl salicylate (with greater levels in Xf-infected plants), and quinic acid (with greater levels in Xf-infected plants).

**Table 4:** **Mean (±SE) sap levels (μg/mL) of phenolic compounds from both non-infected and Xf-infected grapevines with different scion/rootstock combinations**.

**Compound**	**Cabernet Sauvignon**		**Chardonnay**				***F***
	**101-14**	**110R**	**101-14**	**110R**	**RS3**	**SC**	
Caftaric acid	43.2±6.9 c	43.2±7.3 c	81.8±8.8 a	36.3±5.4 c	64.4±9.8 ab	47.9±6.6 bc	5.374^***^
Catechin	335±52 c	411±58 bc	381±64 bc	523±80 ab	657±49 a	481±70 abc	2.945^*^
Epicatechin	293±72 b	397±81 b	487±106 ab	493±79 ab	665±80 a	613±64 a	2.913^*^
Epicatechin gallate	169±26 b	205±31 b	254±35 ab	251±32 ab	318±30 a	316±28 a	3.805^**^
Flavonoid glycoside 1	27.4±7.2 c	37.4±6.0 c	61.8±18.6 abc	54.8±9.1 bc	101±23 a	80.3±11.5 ab	3.793^**^
Flavonoid glycoside 2	35.5±12.0	65.8±18.0	36.5±9.5	33.7±5.6	41.7±10.8	32.5±6.2	1.452
Methyl salicylate	26.2±5.5 b	29.7±5.6 b	40.6±5.5 ab	34.8±5.2 ab	48.6±5.9 a	35.5±4.8 ab	2.516^*^
Procyanidin B isomer 1	67.0±6.6	73.2±6.12	69.2±8.2	73.3±5.5	79.2±7.0	84.6±8.6	0.852
Procyanidin B isomer 2	76.6±13.3	96.3±19.4	94.7±16.3	124±34	131±22	96.4±11.4	1.547
Procyanidin B isomer 3	165±28 b	210±34 b	235±35 ab	226±34 ab	300±25 a	307±32 a	2.917^*^
Procyanidin B isomer 4	40.7±6.8 c	64.1±9.5 c	160±31 ab	124±20 b	185±25 a	146±20 ab	8.761^***^
Procyanidin B1	200±32 d	266±43 cd	299±52 bcd	341±58 abc	452±50 a	440±50 ab	4.086^**^
Procyanidin B2	165±42 c	240±46 bc	328±60 ab	319±56 ab	385±52 ab	440±66 a	3.816^**^
Procyanidin C isomer 1	68.3±10.4 c	75.3±9.5 c	81.4±10.3 bc	96.9±12.0 abc	105±7 ab	116±14 a	3.392^**^
Procyanidin C isomer 2	140±21	166±31	207±53	211±33	269±30	254±37	2.088
Procyanidin C isomer 3	88.8±22.3	147±27	107±32	129±26	180±31	105±11	1.502
Procyanidin C isomer 4	42.2±11.0 c	76.2±18.4 bc	94.2±18.3 ab	93.2±12.6 ab	139±25 a	93.6±10.8 ab	3.766^**^
Procyanidin C1	141±30 c	180±34 bc	215±36 ab	201±28 bc	262±23 ab	290±29 a	3.168^*^
Quinic acid	28.1±2.6 bc	33.8±4.8 ab	24.3±3.0 bc	39.7±5.4 a	31.4±4.8 abc	21.3±2.9 c	2.992^*^
Quinic acid dimer	26.7±2.8	44.8±9.3	23.7±2.9	31.9±5.0	42.7±7.7	27.9±5.5	1.891

The F-test statistic is provided. Different letters next to the means indicate significant differences as determined by LSD tests.

* P < 0.05;

** P < 0.01;

*** P < 0.001.

In Xf-infected plants, only sap levels quinic acid were significantly positively associated with PD symptoms (ρ = 0.346; *P* = 0.049; *N* = 33). Also in Xf-infected plants, sap levels of procyanidin B isomer 2 (ρ = 0.392; *P* = 0.027; *N* = 32), quinic acid dimer (ρ = 0.514; *P* = 0.003; *N* = 32), and quinic acid (ρ = 0.439; *P* = 0.012; *N* = 32) were positively associated with Xf titer. No other significant correlations were observed.

### Selected rootstock effects on root mass and length

**Figure 3:**
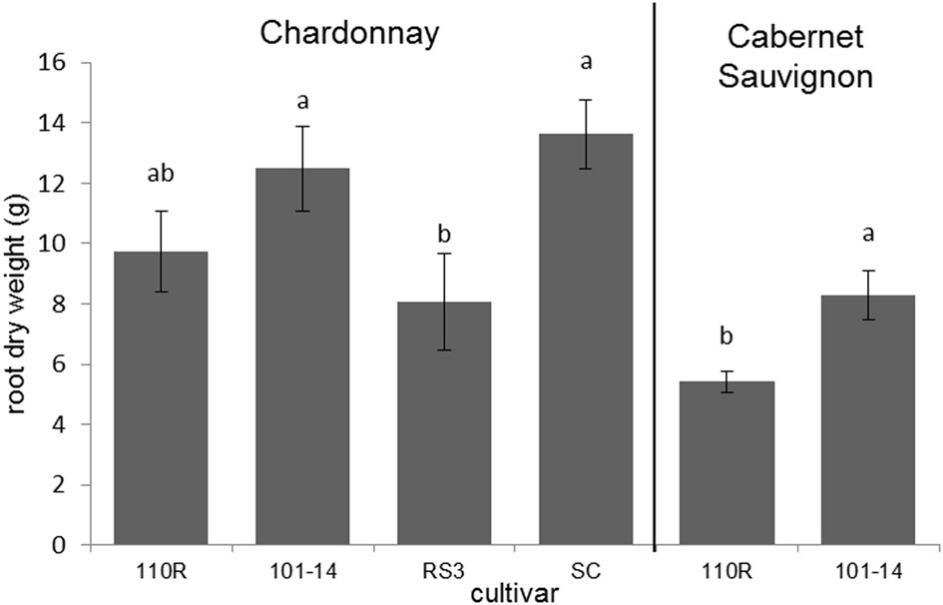
**Root dry weights of Chardonnay or Cabernet Sauvignon grafted to select rootstock cultivars**. Letters represent pairwise differences by LSD for each scion. Bars indicate SE.

The dry root masses of plants consisting of Chardonnay grafted to 101-14 and SC were significantly greater (*F* = 3.549; *P* = 0.033; *N* = 24) than those of plants in which Chardonnay was grafted to RS3 (Figure 3). Likewise, plants in which Cabernet Sauvignon was grafted to 101-14 had significantly greater (*F* = 10.714; *P* = 0.008; *N* = 12) dry root masses than plants which had Cabernet Sauvignon grafted to 110R (Figure 3). Root length did not significantly (*P* > 0.05) differ due to rootstock for either Chardonnay or Cabernet Sauvignon plants. Neither root mass nor length were significantly (*P* > 0.05) correlated with PD severity. However, both root mass (ρ = −0.391; *P* = 0.040; *N* = 28) and root length (ρ = −0.433; *P* = 0.019; *N* = 29) were negatively correlated with Xf titer. Root dry mass was positively associated with levels of epicatechin gallate (ρ = 0.400; *P* = 0.035; *N* = 28), procyanidin B1 (ρ = 0.410; *P* = 0.030; *N* = 28), procyanidin B2 (ρ = 0.465; *P* = 0.013; *N* = 28), procyanidin B isomer 1 (ρ = 0.395; *P* = 0.038; *N* = 28), procyanidin B isomer 4 (ρ = 0.629; *P* < 0.001; *N* = 28), and procyanidin C isomer 2 (ρ = 0.476; *P* = 0.010; *N* = 28). Root mass also was negatively associated with quinic acid dimer levels (ρ = −0.410; *P* = 0.030; *N* = 28). Root length was negatively associated with quinic acid levels (ρ = −0.396; *P* = 0.033; *N* = 29). No other significant associations were observed.

## Discussion

Based on these results, rootstock selection had effects on grapevine scion tolerance to Xf infection. Particular rootstocks, such as 101-14 and 420A, appeared to reduce PD symptom development over the 6 month experiment. However, all of the grapevines remained susceptible to Xf infection and exhibited some PD symptoms. Only Chardonnay on RS3 had significantly greater Xf titers than Chardonnay on other rootstocks. Xf titers did not differ in Cabernet Sauvignon grapevines grafted to different rootstocks.

SC and Freedom might become preferred rootstocks for Chardonnay growers if PD is a concern. However, further evaluation is needed of these rootstocks. For those growing Cabernet Sauvignon, 101-14, 420A, 1103P, and Schwarzmann warrant consideration if PD is a concern, but these also need to be evaluated further. In particular, Schwarzmann had high PD severity when Chardonnay was used as the scion. Of the rootstocks that were grafted to both scions, 101-14 was observed to consistently have moderate to moderate-low PD symptoms. Therefore, future field evaluations observing if rootstocks can impart increased tolerance to Xf infection should include this cultivar.

There was no evidence that having a particular wild *Vitis* spp. in the background of a rootstock conferred increased PD tolerance to the scion. For instance, although both SC and Freedom rootstocks, which had few PD symptoms develop, had *Vitis champinii* in their background, so did RS3, which had severe PD symptoms when Chardonnay was the scion. However, the parent wild species used to develop rootstock cultivars still plausibly have genes that could be associated with PD tolerance, and these findings might be a result of such genes not being inherited by all hybrids.

Regarding links between observed PD tolerance and other traits that these rootstocks reportedly provide, greater resistance to crown gall or drought, as reported by Keller (2010), did not appear to have any relationship with our findings. However, both SC and Freedom, which when used as rootstocks appeared to reduce scion PD symptoms, were bred for nematode resistance (Anwar et al., 2002), and this trait may be indirectly associated with observed imparted scion tolerance to PD. Increased vigor also was observed among the rootstocks which had the fewest PD symptoms, both according to reported grafted scion vigor (Keller, 2010) and root vigor observations from this study. Root vigor also was significantly negatively correlated with Xf titer. This was in agreement with the observations made by Cousins and Goolsby (2011), who also observed rootstocks with fewer PD symptoms tended to have improved vigor.

Scion sap phenolics levels were examined to observe whether they varied among rootstock selections, and whether or not such variations in phenolics could be a potential mechanism behind observed PD symptoms and Xf titers. Rootstock selections did affect scion xylem sap levels of most phenolics. Specifically, sap levels of almost every catechin or procyanidin were significantly lower in grapevines grafted to 101-14, regardless of scion cultivar. This was unexpected as 101-14 had fewer PD symptoms than other cultivars, but previous work observed a complicated relationship between observed sap phenolic levels and Xf infection (Wallis and Chen, 2012; Wallis et al., 2013).

Chardonnay grafted to 101-14 was observed to have greater sap levels of caftaric acid, and this compound plausibly could have by itself improved PD tolerance. Caftaric acid was one of a few phenolic compounds (along with the defense-associated hormone methyl salicylate and quinic acid) to be significantly greater in infected vs. non-infected grapevines. This suggests that caftaric acid was produced as part of an infection-induce coordinated defense response to combat Xf infection. Previous work by Goetz et al. (1999) observed that caftaric acid inhibited a fungal pathogen-produced stilbene oxidase, which had the putative function of detoxifying resveratrol and other plant-produced antibiotic compounds (stilbenoids). It is possible that caftaric acid might act in a similar manner in the case against *Xylella fastidiosa*-detoxifying enzymes, but further investigation is needed.

Yet, caftaric acid in this case was not significantly associated with PD symptoms. Rather, the only phenolic significantly associated with PD symptoms and Xf titers was quinic acid, which had levels positively associated with PD symptoms and Xf titers.

Even if phenolic levels did not affect PD symptom progression or Xf titers at all, changes in xylem sap chemistry due to rootstock might affect grapevine scion tolerance to other diseases, insect pests, or abiotic stresses such as drought. Future studies are warranted to further delve into the ability of rootstocks to affect scion biochemistry and, in turn, examine the potential effects this could have on grapevine scion tolerance to a variety of stressors. Further, levels of phenolics may change over the course of Xf infections (Wallis and Chen, 2012). Expanded studies may want to consider monitoring earlier shifts in phenolics that occur during Xf infections, and how those relate to eventual PD progression. This study was focused only on phenolic levels at 6 months post-infection, as symptoms of PD in this greenhouse experiment did not begin to appear until about 5 months post-inoculation and the objective was to relate existing phenolic levels to PD symptom severity.

In conclusion, analysis of these results indicated that rootstocks have potential to reduce PD progression in Xf-infected scions. Regardless of mechanism, the use of particular rootstocks to impart even a minor increase in PD tolerance to commercial grapevines could provide significant long-term reductions in losses due to PD. The use of PD-tolerance imparting rootstocks may reduce the ability of Xf to move from initial inoculation sites in vines to the canes. If Xf infections are limited to vines at the end of the season, such infected vines might be removed during pruning or the phenomena of “cold-curing” might eliminate the infections (Lieth et al., 2011). Therefore, these results exhibit the promise of using rootstocks to reduce PD severity in locations where it is a major concern. However, before recommendations can be made to growers regarding the selection of particular rootstocks to improve PD tolerance, additional studies are warranted including increasing the number of scions examined as well as performing multiyear field trials.

## Author contributions

Christopher M. Wallis designed this project. Christopher M. Wallis performed the plant inoculations and evaluated PD symptoms. Christopher M. Wallis and Anna K. Wallingford harvested plant samples and analyzed phenolics. Anna K. Wallingford and Jianchi Chen performed Xf titer analyses. Christopher M. Wallis and Anna K. Wallingford performed statistical analyses. Christopher M. Wallis wrote the manuscript, with input and editing performed by Anna K. Wallingford and Jianchi Chen.

### Conflict of interest statement

The authors declare that the research was conducted in the absence of any commercial or financial relationships that could be construed as a potential conflict of interest.
